# EXCURSION FROM HOME AND ECOLOGICAL PAIN IN OLDER ADULTS WITH KNEE PAIN

**DOI:** 10.1093/geroni/igz038.1929

**Published:** 2019-11-08

**Authors:** Mamoun T Mardini, Parisa Rashidi, Subhash Nerella, Dottington Fullwood, Duane Corbett, Sanjay Ranka, Todd M Manini

**Affiliations:** University of Florida, Gainesville, Florida, United States

## Abstract

Background: Pain is an essential factor in limiting life-space mobility. Ecological momentary assessment (EMA) is crucial to understanding pain intensity and frequency. This study evaluated a customized smartwatch app for daily ecological pain reporting and GPS (Global Positioning System) coordinates collection to understand the impact of pain on daily excursion from home in older adults who report knee pain. Methods: Participants (n=14, 73.2 +/- 5.4 yrs, 64% female) wore a smartwatch with a customized app called Patient Reported Outcome of Mood, Pain, and fatigue (PROMPT) for 6.5 (4.0) days. Participants were prompted in their free-living environment about their pain intensity (range 0-10) in the morning, afternoon and evening. Additionally, GPS data were collected at 15 min intervals throughout the day. Geodesic distance was used to calculate the distance from the home address. Daily pain values were binned into high and low levels to compare to maximum daily excursions. Results: Individuals with average daily pain > 2 traveled 4.1 fewer miles than those individuals reporting pain 2 exceeded a distance of 5 miles compared to 17.9% of those individuals reporting pain <= 2 (X2=6.89, p < 0.05). Conclusion: In older adults, higher level of knee pain is associated with a decline in life-space mobility. Using custom designed smartwatch applications provides new opportunity to investigate how pain impacts community mobility.

